# Enrichment analysis and chromosomal distribution of gout susceptible loci identified by genome-wide association studies

**DOI:** 10.17179/excli2023-6481

**Published:** 2023-11-14

**Authors:** Mostafa Saadat

**Affiliations:** 1 Department of Biology, School of Science, Shiraz University, Shiraz 71467-13565, Iran

**Keywords:** chromosome, enrichment analysis, gout, gene ontology

## Abstract

Gout is an inherited and common inflammatory arthritic disease. Many researchers will identify polymorphic loci of gout susceptibility by conducting genome-wide association studies (GWAS). In the present study, the enrichment analysis and chromosomal distribution were performed using predicted polymorphic loci associated with gout risk. The polymorphic loci associated to gout were obtained from the GWAS database. Overall, this database contains 64,806 gout patients and 2,856,174 controls. Gene ontology functional annotation and Kyoto Encyclopedia of Genes and Genomes (KEGG) pathway analysis were performed by using the Enrichr online server. A total of 110 common polymorphic protein-coding loci associated with gout risk were identified and included in the analysis. The results of the KEGG analysis showed that the gout-associated loci were mainly related to ABC transporters, endocrine and other factor-regulated calcium reabsorption, and gastric acid secretion pathways. The gene ontology analysis showed that the biological processes of the gout-associated loci were vascular transport, transport across the blood-brain barrier, positive regulation of transporter activity, and positive regulation of transcription by RNA polymerase II. The top cellular component was the external side of the apical plasma membrane. Statistical analysis revealed that the human chromosome segments 1q22, 4p16.1, 6p21.1-p21.2, 11q13.1-q13.2, 12q13.11-q13.3, and 12q24.1 had significantly bearing higher numbers of gout susceptibility loci.

## Introduction

Gout is a common inflammatory arthritic disease. It is caused by the deposition of monosodium urate crystals in articular and non-articular structures. Hyperuricemia (elevated blood urate) is the major risk factor for the development of gout. It is often associated with other conditions such as hypertension, cardiovascular disease, diabetes, dyslipidemia, obesity, chronic kidney disease, and kidney stones. Epidemiological studies have reported that gout has an incidence of 0.6-2.9 per 1000 person-years and a prevalence of 0.68-3.90 % in adults (Dalbeth et al., 2021[6]). 

Several familial aggregation studies and comparisons of monozygotic and dizygotic twins for hyperuricemia, renal clearance of urate, and gout have shown that these traits are multifactorial, with significant heritability (Emmerson et al., 1992[10]; Wilk et al., 2000[51]; Bleyer and Hart, 2006[3]; Voruganti et al., 2009[50]; Krishnan et al., 2012[19]; Kuo et al., 2015[21]). This means that both genetic and non-genetic environmental factors are involved in the pathogenesis of these disorders. 

Many researchers will identify the genetic elements of gout susceptibility by conducting genome-wide association studies (GWAS) (Sulem et al., 2011[44]; Lai et al., 2012[22]; Shin et al., 2012[41]; Köttgen et al., 2013[18]; Li et al., 2015[26]; Matsuo et al., 2016[30]; Nakayama et al., 2017[31], 2020[32]; Chen et al., 2018[4]; Jing et al., 2018[14]; Lee et al., 2019[24], 2022[23]; Kawamura et al., 2019[17]; Tin et al., 2019[47]; Backman et al., 2021[1]; Dönertaş et al., 2021[8]; Fitzgerald et al., 2022[11]; Jiang et al., 2021[13]; Sandoval-Plata et al., 2021[40]; Toyoda et al., 2022[49]; Lin et al., 2023[28]; Sumpter et al., 2023[45]) or by examining the association between common genetic polymorphisms and gout risk in case-control studies (Dong et al., 2015[9]; Lee et al., 2017[25]; Zou et al., 2018[53]; Kawaguchi et al., 2021[16]).

Today, enrichment analysis (also called gene set enrichment analysis, functional enrichment analysis, or pathway enrichment analysis) is a popular method for analyzing gene/protein sets that is essentially developed using complex statistical analysis methods. These analyses are used to identify classes of genes or proteins that are overrepresented in a large set of genes or proteins. In other words, enrichment analysis is a statistical method for determining enriched or depleted groups of genes or proteins (Subramanian et al., 2005[43]). 

In the present study, the enrichment analysis was performed using predicted polymorphic loci associated with gout risk, and the chromosomal distribution of the associated loci was constructed to identify the non-random chromosomal segments associated with gout.

## Methods

### Search for gout associated loci

The polymorphic loci associated with gout were retrieved from the Genome Wide Association Studies (GWAS) database (https://www.ebi.ac.uk/gwas) on August 10, 2023 using gout as a keyword. 

### Enrichment analysis

Because enrichment analysis involves complex statistical analysis, it requires a computer program. Several tools are available to perform the analysis. One of these computational analysis tools is the web-based Enrichr. Enrichr contains various data sets, such as pathways and protein interactions, gene ontologies, and gene expression in different tissues and cells.

The pathway enrichment analysis and gene ontology analysis were analyzed using the Enrichr online server ([link:http://maayanlab.cloud/Enrichr*http://maayanlab.cloud/Enrichr]) (Chen et al., 2013[5]; Kuleshov et al., 2016[20]; Xie et al., 2021[52]). For pathway enrichment analysis, the KEGG 2021 human database was selected to retrieve pathways (Kanehisa and Goto, 2000[15]; Bindea et al., 2009[2]; Jassal et al., 2020[12]). Gene Ontology (GO) enrichment analysis was performed, including those associated with molecular functions, cellular components, and biological processes (Kanehisa and Goto, 2000[15]). Adjusted p-value was used to exclude the influence of multiple comparisons in p-values. Adjusted p<0.05 was considered statistically significant.

### Randomness of chromosomal location

The chromosomal location of the loci associated with susceptibility to gout was extracted from the OMIM database (https://www.omim.org). The non-randomness of the chromosomal distribution of these loci was statistically evaluated using the method of Tai et al. (1993). The relative nucleotide length of each chromosomal segment to the whole haploid genome was determined using data from the Ensembl Genome Browser (https://asia.ensembl.org/Homo_sapiens/Location/Genome?db=core). To reduce of false positives (type I statistical error reduction), a p<0.001 was considered statistically significant.

## Results

We found extracted data from 22 published GWAS studies in the database (Sulem et al., 2011[44]; Lai et al., 2012[22]; Shin et al., 2012[41]; Köttgen et al., 2013[18]; Li et al., 2015[26]; Matsuo et al., 2016[30]; Nakayama et al., 2017[31], 2020[32]; Chen et al., 2018[4]; Jing et al., 2018[14]; Lee et al., 2019[24], 2022[23]; Kawamura et al., 2019[17]; Tin et al., 2019[47]; Backman et al., 2021[1]; Dönertaş et al., 2021[8]; Fitzgerald et al., 2022[11]; Jiang et al., 2021[13]; Sandoval-Plata et al., 2021[40]; Toyoda et al., 2022[49]; Lin et al., 2023[28]; Sumpter et al., 2023[45]). Overall, this database contains 64,806 gout patients and 2,856,174 controls. A total of 245 significant associations were initially extracted. For some genes, more than one genetic polymorphism was investigated. Only protein-coding genes were included in the present analysis. Finally, a total of 110 common polymorphic protein-coding loci associated with gout risk were identified and included in the analysis (Table 1(Tab. 1)).

The results of the KEGG analysis are shown in Table 2(Tab. 2). The associated loci were mainly related to ABC transporters (*CFTR*, *ABCC9*, *ABCC8*, *ABCG1*, and ABCG2), endocrine and other factor-regulated calcium reabsorption (*PRKCA*, *ATP1A4*, *BDKRB2*, and *VDR*), and gastric acid secretion (*KCNQ1*, *PRKCA*, *CFTR*, and *ATP1A4*) pathways.

The gene ontology (GO) analysis consisted of three functional parts, including biological process (BP), cellular component (CC), and molecular function (MF). The top four biological processes were vascular transport (GO: 0010232), transport across blood-brain barrier (GO: 0150104), positive regulation of transporter activity (GO: 0032411), and positive regulation of transcription by RNA polymerase II (GO: 0045944). The top cellular component was the external side of the apical plasma membrane (GO: 0098591) with two *ABCG2*, and *SLC38A1* genes. There was no statistically significant gene ontology analysis for the molecular functions (Table 2(Tab. 2)).

Shared polymorphic loci between gout and selected traits, based on GWAS Catalog 2023 was investigated. The results were summarized in Table 3(Tab. 3). Chronic kidney disease, kidney stones, type 2 diabetes, fasting glucose, triglyceride levels, metabolic syndrome, coronary artery disease, diastolic and systolic blood pressure, resistant hypertension, systemic lupus erythematosus, alcohol dependence, schizophrenia, rate of cognitive decline in Alzheimer's disease, Alzheimer's disease, bipolar disorder or major depressive disorder, COVID-19 (critical illness vs population or mild symptoms), severe COVID-19 infection, COVID-19 (hospitalized vs population) were selected traits which had shared polymorphic loci with gout.

Of 110 potentially gout-associated polymorphic loci, 3 (*MUC1*, *THBS3*, and *TRIM46*), 3 (*CLNK*, *SLC2A9*, and *WDR1*), 5 (*ABCG2*, *DMP1*, *MEPE*, *PKD2*, and *SPP1*), 4 (*PRSS16*, *ZSCAN31*, *CARMIL1*, and *H4C5*), 8 (*CDC42BPG*, *MAP3K11*, *NRXN2*, *OVOL1*, *RPS6KA4*, *SLC22A11*, *CNIH2*, and *POLD3*), 4 (*SLC38A1*, *VDR*, *INHBC*, and *R3HDM2*), and 5 (*CUX2*, *ACAD10*, *ALDH2*, *NAA25*, and* TRAFD1*) genes were located on the human 1q22, 4p16.1, 6p21.1-p21.2, 11q13.1-q13.2, 12q13.11-q13.3, and 12q24.1 chromosome segments, respectively. These chromosomal distributions are not random (Table 4(Tab. 4)). There was no statistical evidence that the other gout-associated loci non-randomly distributed on the chromosomes. 

## Discussion

This study used data available in the GWAS database on polymorphic loci associated with gout risk. A total of 110 common polymorphic protein-coding loci associated with gout risk were identified and included in the analysis. Enrichment analysis was then performed using the Enrichr tool. The present study showed that in the gene ontology analysis, BP mainly focuses on vascular transport, transport across the blood-brain barrier, positive regulation of transporter activity, and positive regulation of transcription by RNA polymerase II, CC mainly focuses on the external side of the apical plasma membrane.

Previously, Qiu and colleagues reported differentially expressed genes (DEGs) in gout using the GEO database (Qiu et al., 2022[36]). They reported that the results of gene ontology analysis of the DEGs were mainly enriched in immune and inflammatory response, cytokine and growth factor activities; also KEGG pathway analysis showed that the DEGs were mainly related to chemokine signalling pathway and cytokine-cytokine receptor interaction (Qiu et al., 2022[36]). It should be noted that the present results are not only not similar to those of Qiu et al., but also quite different. There is no commonality in the results of the enrichment analysis between the Qiu study and the present study. Among the polymorphic genes associated with gout, there is no gene involved in the immune system. At present, it is very difficult to interpret this discrepancy. However, some suggestions can be made. First, they used only one data set, whereas we used all available data sets. Second, the analysis of differentially expressed genes (DEGs) was based on a very small sample size (12 participants including 6 gout patients and 6 healthy controls), whereas the GWAS data were obtained from very large samples. Third, the subjects in Qiu's study were all Chinese males, whereas the present study used data from both sexes belonging to different ethnic groups. Finally, the differentially expressed genes and the polymorphic genes are two different sets of genes that are involved in the pathogenesis of gout.

As mentioned in the introduction, gout is often associated with other conditions such as hypertension, cardiovascular disease, diabetes, dyslipidemia, chronic kidney disease, and kidney stones (Dalbeth et al., 2021[6]). The present study showed that these traits shared polymorphic loci with gout (Table 3(Tab. 3)). Surprisingly, both susceptibility to COVID-19 and mortality due to COVID-19 shared polymorphic protein-coding genes with gout (Table 3(Tab. 3)). It should be noted that there are significant associations between gout and both susceptibility to COVID-19 and COVID-19-related death (Dalbeth and Robinson, 2021[7]; Peng et al., 2022[35]; Nissen et al., 2022[33]; Topless et al., 2022[48]). An association between low serum urate concentrations and the risk of neurodegenerative diseases, such as Parkinson's disease and Alzheimer's disease, has been reported previously (Li et al., 2017[27]; Singh and Cleveland, 2019[42]). A meta-analysis of four cohort studies reported that gout and hyperuricemia might reduce the risk of AD (Pan et al., 2021[34]). Interestingly, Alzheimer's disease shared polymorphic loci with gout (Table 3(Tab. 3)). Previously, it has been reported that genes associated with the risk of Alzheimer's disease are not randomly distributed on human chromosomes. One of the human chromosomal segments carrying Alzheimer's disease-associated genes is 6p21 (Saadat, 2016[38]). Interestingly, the present study indicated that 6p221-p22.2 chromosome segment which is located in the vicinity of 6p21 and obviously had linkage disequilibrium with each other, carries gout associated loci (Table 4(Tab. 4)). 

The present finding of non-random chromosomal distribution of gout-associated loci is similar to the non-random distribution of some other disease-associated (such as breast and gastric cancers, Alzheimer's disease) genes on human chromosomes, which supports non-random distribution of genes in the construction of human chromosomes (Saify and Saadat, 2012[39]; Saadat, 2014[37], 2016[38]; Mahjoub and Saadat, 2018[29]). 

The present findings suggest the possibility of designing and developing a laboratory diagnostic test method using the genetic variations on the human chromosome segments 1q22, 4p16.1, 6p21.1-p21.2, 11q13.1-q13.2, 12q13.11-q13.3, and 12q24.1 for use in mass screening programs to identify individuals at high risk for developing gout.

## Conflict of interest

The author declares no conflict of interest.

## Figures and Tables

**Table 1:**
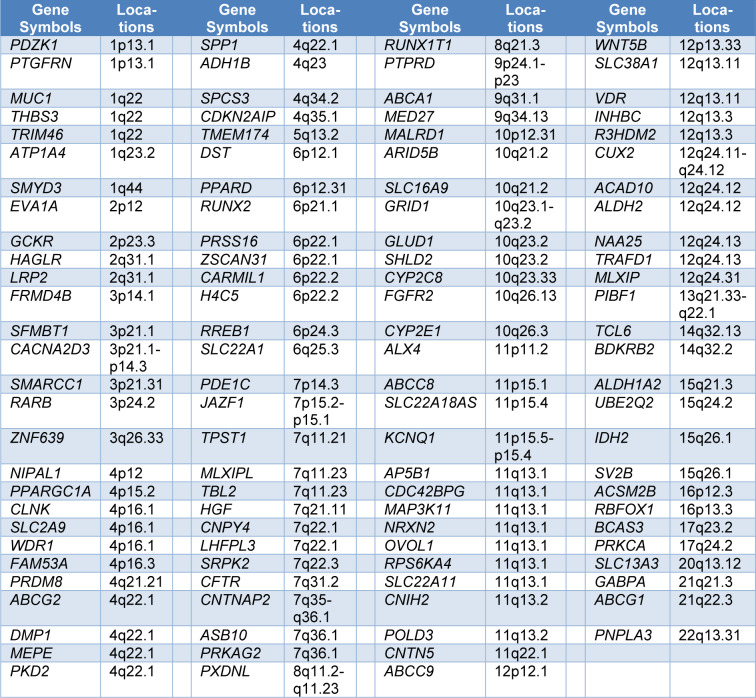
Polymorphic genes associated with the risk of gout and their cytogenetic locations

**Table 2:**
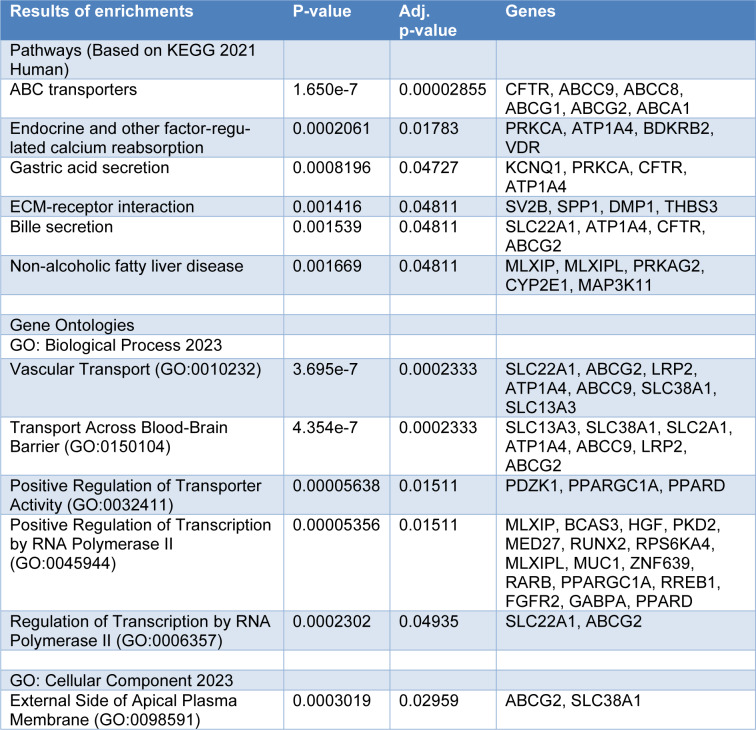
Significant findings of pathways and gene ontologies

**Table 3:**
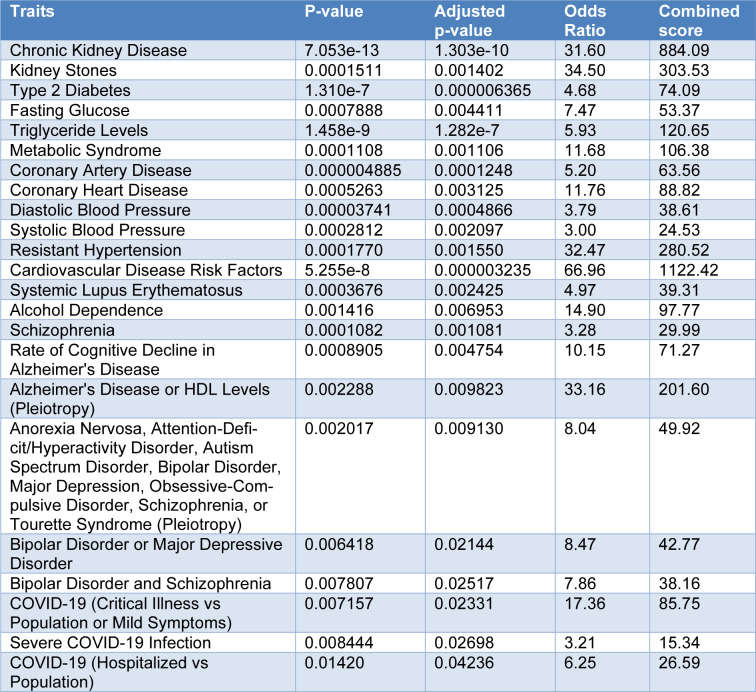
Shared polymorphic loci between gout and selected traits

**Table 4:**
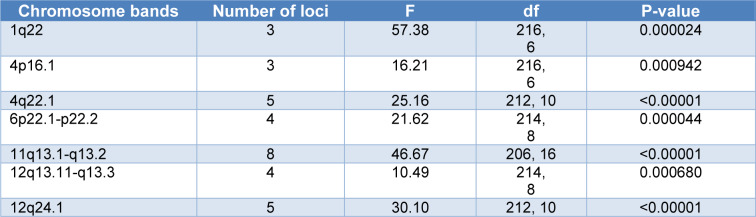
Non-random distribution of gout susceptible loci on human chromosomes
